# Comparative effectiveness of aerobic exercise versus *Yi Jin Jing* on ovarian function in young overweight/obese women with polycystic ovary syndrome: study protocol for a randomized controlled trial

**DOI:** 10.1186/s13063-022-06377-8

**Published:** 2022-06-03

**Authors:** Jing Zhao, Antonnette Ketlhoafetse, Xiangyun Liu, Yang Cao

**Affiliations:** 1 Key Laboratory of Exercise and Health Sciences of Ministry of Education, Shanghai University of Sport, 188 Hengren Road, Shanghai, 200438 China; 2 Chinese Medicine Yueyang Hospital of Integrated Traditional Chinese Medicine and Western Medicine, Shanghai University of Traditional, 110 Ganhe Road, Shanghai, 200438 China

**Keywords:** Polycystic ovary syndrome, Aerobic exercise, Yi Jin Jing, Traditional chinese exercise

## Abstract

**Background:**

Polycystic ovary syndrome (PCOS) is the most common heterogeneous endocrine disease among women of childbearing age, characterized by androgen excess and ovarian dysfunction. Aerobic exercise is an important solution used to manage PCOS, due to its multiple benefits. *Yi Jin Jing* is an important component of traditional Chinese exercise (TCE), based on the root of traditional Chinese medicine theory (TCM), which focuses on keeping the body as a whole in a harmonious state. However, to date there is no literature on the benign effects of *Yi Jin Jing* on PCOS. The primary purpose of this protocol is to assess the effectiveness of aerobic exercise versus *Yi Jin Jing*, on the management of ovarian function in young overweight/obese women with PCOS.

**Methods:**

The study will conduct a controlled randomized, superiority trial with three-arm parallel groups, recruiting 90 women diagnosed with PCOS, ages between 18 and 35 years, with a body mass index (BMI) ≥ 23 kg/m^2^. Women will be randomly assigned to either control group (combined oral contraceptives) or one of the intervention groups (*Yi Jin Jing* group or aerobic exercise group) with an allocation rate of 1:1:1. After randomization, the intervention will be conducted within a 12-week period. The primary outcome would be anti-Mullerian hormone (AMH) level; the secondary outcomes would be biochemical profiles, ovarian volume, antral follicle count, BMI, menstrual frequency, and homeostasis model assessment of insulin resistance (HOMA-IR). Outcome measures would be collected during baseline and end of treatment. Reporting of the study will follow the Standard Protocol Items: Recommendations for Interventional Trials (SPIRIT) statement.

**Discussion:**

This proposed study will be the first randomized clinical trial to evaluate the comparative effectiveness of aerobic exercise versus *Yi Jin Jing* on the management of ovarian function in young overweight/obese women with PCOS. The results may provide a new evidence-based management strategy for young women with PCOS.

**Trial registration:**

Chinese Clinical Trial Registry ChiCTR1900022385. Registered on 9 April 2019

**Supplementary Information:**

The online version contains supplementary material available at 10.1186/s13063-022-06377-8.

## Background

Polycystic ovary syndrome (PCOS) is the foremost endocrinopathy which affects 6–10% of the reproductive stages in women [1]. PCOS is characterized by androgen excess and ovarian dysfunction [2] and is further aggravated by hyperinsulinemia [3], thus causing a number of reproductive and metabolic dysfunctions. The main clinical features include anovulation, menstrual irregularity, infertility, acne, and metabolic disorders [2, 4, 5], affecting diagnosed limiting women lifespan [6] and reducing health-related quality of life. Presenting signs and symptoms are heterogeneous and could change with aging; reproductive function disorders are the primary disturbances in younger women with PCOS [7–9]. About 60% PCOS women are overweight or obese [10], and excess weight could significantly worsen reproductive features for PCOS [11], as well as weakening the effectiveness of fertility treatment and increasing the risk of pregnancy complications [12]. In 2005, the annual cost on reproductive-aged PCOS women in the USA was up to $4.36 billion [13], resulting in a tremendous economic burden to families and the society as a whole. Thus, improving ovarian function is crucial for young overweight/obese women with PCOS.

Lifestyle modification (diet and exercise) is recommended as the first-line management for PCOS to achieve effective weight management and to optimize hormonal profiles, ultimately improving quality of life [14, 15]. In relation to diet, exercise has equal and sustainable effect in maintenance of weight loss, improvement of menstrual status, and ovulation in overweight/obese women with PCOS [16]. While dietary program is at the expense of muscle mass [16], exercise could reduce more fat mass, retain lean muscle mass, and improve body composition [17, 18], suggesting the optimal role exercise plays in lifestyle management for PCOS. Al-Eisa et al. [19] found that 12-week aerobic training can significantly improve reproductive function by decreasing body mass index (BMI), anti-Mullerian hormone (AMH), and antral follicle count in PCOS group, while increasing follicle-stimulating hormone (FSH) and estradiol (E_2_). Aerobic exercise could significantly reduce the number of follicles developments between 2 and 9 mm and improve ovarian morphology [20]. Aerobic exercise could improve quality of life of women with PCOS [21]. An updated meta-analysis consisting of 18 studies, with total of 1978 participants showed that exercise intervention may improve pregnancy rates in women with reproductive health problems [22]. However, another recent meta-analysis including 14 studies which evaluated a total of 617 women with PCOS pointed out that the impact of exercise interventions on ovarian function remains ambiguous, without sufficient evidence to quantify the effect of exercise on ovulation quantitatively of affected women [23]. The conflicting evidence suggests the need for further studies on the effect of exercise intervention on ovarian function of women with PCOS.

According to traditional Chinese medicine (TCM) theory, kidney deficiency is viewed as the root problem in PCOS [24], kidney dominates the function of “kidney-Tian Gui-Chong Ren-uterus axis” [24]. The dysfunction of hypothalamic-pituitary-ovary (HPO) axis plays a role on pathogenesis of PCOS, resulting in increased gonadotrophin releasing hormone and luteinizing hormone (LH), then impacting ovarian androgen synthesis and folliculogenesis [2]. The “kidney-Tian Gui-Chong Ren-uterus” axis of TCM is similar to HPO axis in modern medicine [25]. Where the function of the kidney resembles  that of the hypothalamus [24], the function of *Tian Gui* resembles that of the sex hormone [26], and the function of *Chong* meridian and *Ren* meridian resemble that of the pituitary gonadotropin [24]. Thus, the kidney deficiency could lead to disturbance of *Tian Gui* [27] and disharmony of *Chong* and *Ren* meridians [26].

Furthermore, overweight/obese PCOS women are closely related to phlegm-dampness constitution [28, 29]; phlegm-dampness could block meridians and causes Qi stagnation and blood stasis, aggerating a number of reproductive dysfunctions.

*Yi Jin Jing*, which dates back to ancient China, consists of soft and stretching body movements, breath control, and meditation [30]. Practicing *Yi Jin Jing* could achieve harmonious integration of body and mind, by attaining a relaxed and deep focused state. *Yi Jin Jing*, as an important part of traditional Chinese exercise (TCE), has a theoretical root on TCM, having a dual nature of exercise and medical treatment [31]. *Yi Jin Jing* training emphasis on waist movement thus could strengthen the kidney through waist movement exercises, and the waist is termed as “the house of kidney” from the TCM theory [31]. *Yi Jin Jing* could also stimulate the *Chong* and *Ren* meridians through flexion and extension movements, balancing of the Yin and Yang, and harmonizing Qi [31, 32] to attain the holism and benign effects.

Hong et al. [33] discovered that *Yi Jin Jing* may balance level of estrogen and progesterone, reduce the level of prostaglandins PGF2a, and improve primary dysmenorrhea symptoms, while Chen et al. [34] found that 3 months of *Yi Jin Jing* training could improve sex hormones levels of FSH, LH, and testosterone (T) and reduce the proportion of E_2_/T in elderly men, hinting the benign role of *Yi Jin Jing* on reproductive health. *Yi Jin Jing* could reduce BMI and fat mass and improve lean muscle [35], helping to improve phlegm-dampness constitution. In conclusion, *Yi Jin Jing* could be used to manage PCOS-related symptom by addressing both manifestation (phlegm-dampness) and root cause (kidney deficiency). To our knowledge, no studies investigating the effect of *Yi Jin Jing* in women with PCOS have been conducted. Therefore, we intend to see the effect of *Yi Jin Jing* in PCOS and comparative effectiveness of aerobic exercise versus *Yi Jin Jing* on the reproductive health in young overweight/obese women with PCOS, proving evidence-based solution for feasibility and executing a new convenient mean for managing PCOS.

## Methods/design

### Objectives

The main objective of this study is to evaluate the comparative effectiveness of aerobic exercise versus *Yi Jin Jing* on ovarian function in young overweight/obese women with PCOS.

### Study design

The study is designed as a controlled randomized, superiority trial with three-arm parallel groups. Reporting of the study will follow the Standard Protocol Items: Recommendations for Interventional Trials (SPIRIT) statement (Additional file 1). A total number of 90 diagnosed PCOS patients will be recruited from Yue yang Integrated Chinese and Western Medicine Hospital, Affiliated to Shanghai University of Traditional Chinese Medicine. Patients will be recruited from the gynecologist ward through fliers, posters, and gynecologist recommendations. Patients will be randomly assigned to two intervention and control group with an allocation ratio of 1:1:1. After randomization, the intervention groups will undergo either *Yi Jin Jing* or aerobic exercise training for 12 consecutive weeks and the control group with no training intervention but take oral contraceptives (OCPs). Post-test measurements will be conducted based on the primary and secondary outcomes in comparison to the baseline measures.

### Study setting

All the medical tests will be conducted in the Yue yang Integrated Chinese and Western Medicine Hospital, Affiliated to Shanghai University of Traditional Chinese Medicine. The aerobic exercise and the *Yi Jin Jing* intervention will be conducted at Shanghai University of Sport.

### Inclusion criteria

- (1). Women aged between 18 and 35 years [36, 37]
- (2). Diagnosed with PCOS using the Rotterdam criteria, which at least two of the following are present: oligo-ovulation or anovulation, clinical and/or biochemical signs of hyperandrogenism, and polycystic ovaries as defined by ultrasonography, which is presence of 12 or more follicles in each ovary measuring 2–9 mm in diameter, and/or increased ovarian volume (> 10 mL) [38]
- (3). BMI ≥ 23 kg/m^2^.

### Exclusion criteria

- (1). Known disorders that mimic the PCOS, such as congenital adrenal hyperplasia, androgen-secreting tumors, and Cushing’s syndrome [38]
- (2). Cardiovascular diseases and thrombotic diseases.
- (3). Acute or chronic hepatitis or nephritis.
- (4). Taking medications known to affect ovarian function within the past 3 months.
- (5). Any pulmonary or musculoskeletal diseases that could be impaired by exercise.
- (6). Participating or having regular exercise training during the past 3 months.
- (7). Patients suffer from mental problems.

### Interventions

Aerobic exercise intervention will be conducted on stationary bikes (Lode Excalibur Sport Lode, BV, Groningen, The Netherlands), for 50 min with an intensity level of 65–75% maximum heart rate, 3 times a week (one exercise session for every 2 days), accumulating to 150 min every week [14, 39, 40]. The sessions will include 5-min warm-up and cool down protocols before and after exercise. The 5-min warm-up protocol consists of joint movement, leg stretching, and step movements. The 5-min cool down protocol include walking slowly and stretching the major muscle groups of the body. Aerobic exercise will be conducted at Shanghai University of Sports under the supervision of an accredited physical trainer.

*Yi Jin Jing* group will conduct the *Yi Jin Jing* practice 30 min, 5 times a week [34]; the sessions will also include 5-min warm-up and cool down protocols before and after *Yi Jin Jing* practice. Practice will be conducted at Shanghai University of Sport with the guidance of an experienced *Yi Jin Jing* instructor who has been teaching *Yi Jin Jing* for 5 years.

Control group will take OCPs after randomization. OCPs are the first-line pharmacologic therapy for patients with polycystic ovary syndrome who are not trying to conceive [41, 42]. Diane-35, a kind of OCPs containing 2-mg cyproterone acetate and 35-μg ethinyl estradiol, is the first choice for the management of PCOS patients not seeking fertility in China [43]. The control group will take Diane-35 once daily at the same time from the 5th day of menstruation or withdrawal bleeding for a period of 21 days and for 3 menstrual cycles.

**Fig. 1:**
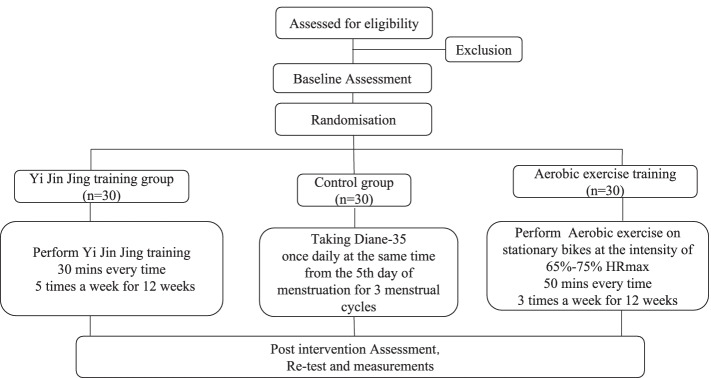
Study flow chart

The study flow chart is shown in Fig. 1. We provide the control group with general exercise recommendations. For ethical reasons, we also provide them with videos of the aerobic exercise and *Yi Jin Jing*. Since exercise in the control group was not monitored, it was assumed that subjects in the control group would have no additional physical activity.

#### Adverse events

If serious injuries occur in the aerobic exercise group or the *Yi Jin Jing* group, such as a severe joint injury that makes it impossible to continue aerobic exercise or *Yi Jin Jing* intervention, it should be reported as an adverse event, and these subjects will stop aerobic exercise or *Yi Jin Jing* intervention and receive the usual medication, which is taking Diane-35. In case of adverse events, the study will have its own physical trainers and specialized practitioners to ensure proper measures are taken in cases of adverse events. Monitoring of adverse events for the aerobic exercise and *Yi Jin Jing* during the trial will include acute pain and heart rate. Therefore, adverse events occurring are estimated to be low.

Side effects of taking Diane-35 including headaches, nausea, weight gain, breast tenderness, and loss of libido will be monitored. If side effects occur, the drug should be stopped immediately for observation and the timing of continued use should be determined by the doctor. Serious adverse reactions will be reported to the ethical committee and other reactions that are thought to be causally associated with the intervention will be managed and recorded in the study.

If a subject suffers a sports injury, such as an ankle injury or muscle strain, during aerobic exercise or *Yi Jin Jing* training, the investigator will manage the acute phase of the sports injury according to the “PRICE” principle. PRICE stands for Protect, Rest, Ice, Compression, and Elevation. After the 48-h acute period, the investigator will provide the subject with appropriate free physiotherapy, such as muscle strength training and functional training. For the control subjects taking oral contraceptives, any side effects of the pill will be adjusted by the doctor and the cost of the examination and medication will be borne by the subject. Exercise and dietary advice and education will continue to be provided to all subjects after the 12-week study.

#### Adherence and compliance

Prior to allocation, all participants will undergo a face-to-face education program with a doctor. All patients are assigned to smart phone app support. These steps are taken to ensure adherence. Control group patients will be asked to send monthly self-monitored information regarding their medication usage and any side effect via their phones for 3 months. Adherence and compliance will be determined from supervised exercise session attendance. Data from participants with less than 75% adherence will be included in the intention to treat analysis.

#### Study measurements

Prior and post to intervention, the following will be measured. Transvaginal ultrasound scan will be performed during the early follicular phase (cycle days 3–5) to document ovary morphology, including antral follicle count and ovarian volume. Fasting blood will be taken for 2–5 days during spontaneous menstrual cycle to check biochemical profiles, including AMH, LH, FSH, T, E_2_, sex hormone binding globulin (SHBG), dehydroepiandrosterone sulfate (DHEA-S), fasting insulin (FINS), and fasting blood glucose (FBG) level. Free androgen index (FAI = T × 100/SHBG) and homeostasis model assessment of insulin resistance (HOMA-IR = FINS × FBG /22.5) will be calculated. All ultrasound and blood tests were performed at the medical laboratory of Yueyang Hospital. All participants will receive a menstrual diary to record menstrual bleedings, menstrual frequency which will be calculated by dividing the number of menstrual bleedings by 3 (ordinal variables: “0,” “1/3,” “2/3,” “1”). Anthropometric indicators including weight, height, and BMI will be measured.

#### Outcome measurements

The primary outcome is serum AMH level. AMH is the best marker of the ovarian function; it could be noticed as a suitable hormonal marker of the ovarian follicular count and as a diagnostic marker for ovarian hyperandrogenism [44–46]. Therefore, we choose AMH as the primary outcome.

Secondary outcomes include:

- (1). Menstrual frequency.
- (2). Biochemical profile including FSH, LH, T, E_2_, SHBG, DHEA-S, FAI.
- (3). Antral follicle count and ovarian volume.
- (4). BMI
- (5). HOMA-IR

#### Participant timeline

**Fig. 2:**
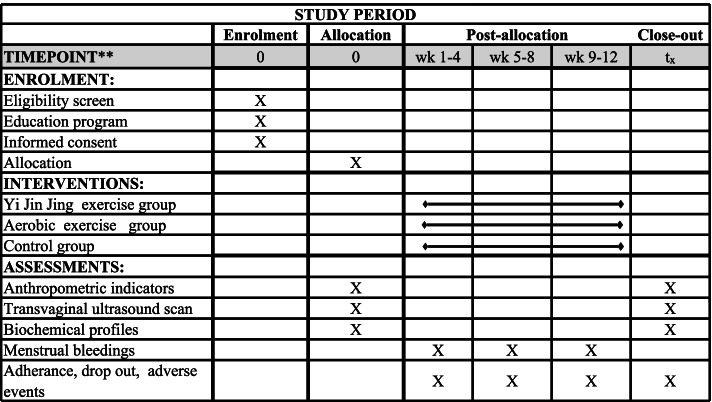
Participant timeline

Participant timeline is described in Fig. 2.

#### Sample size

AMH is the target effect, according to a similar research by Moran [47], where the mean AMH level before intervention was 59.1pmoL/L, standard deviation was 20.5pmoL/L, and AMH declined by 13.2pmoL/L in response to exercise. We require 25 participants per group to achieve a power of 80%, two-sided *α* = 0.05. With an approximate attrition rate of 20%, we will recruit 90 participants in total with 30 participants per group.

#### Recruitment

Enough patients will be recruited through posters, word of mouth, and recommendations from gynecologists.

#### Randomization and concealment

Eligible participants will be randomly assigned into the control group or the intervention group (aerobic exercise group or *Yi Jin Jing* group) with an allocation rate of 1:1:1. The randomization procedure will be conducted by an independent statistician using a computerized program. The allocation sequence will be stored in a sealed envelope and will not be announced until the baseline measurements have been completed.

## Blinding

Due to the characteristic of exercise intervention, all participants and the aerobic exercise supervisor and *Yi Jin Jing* instructor will not be blinded. But the staff who undertake outcome measuring and statistician who undertakes data processing will be blinded to group allocation.

### Data collection and management

Study-related information, such as participant’s identity, the data collected relating to the study, and medical records, will remain confidential. Case report forms (CRFs) will be completed on paper forms. With regard to self-reported measures, participants will monitor their menstrual cycles using a menstrual diary.

### Data monitoring

The data will be recorded in the CRFs. The CRFs will be filled out truly and accurately. Confidentiality of participants will be protected and guaranteed by storing the hard copies of the data collection forms in locked cabinets in the principal investigator’s office. Access to electronic clinical report forms stored in the excel spreadsheets data file will be password-encrypted and restricted to the principal investigators. The blinded analyst will be provided with a de-identified dataset to preserve confidentiality. This study is supervised by Shanghai University of Sport, and the Office of Postgraduate Affairs of Shanghai University of Sport constitutes the data monitoring committee to monitor and review the data.

### Statistical methods

The intention-to-treat principle will be used in the statistical analysis. We will use multiple imputation, based on 5 replications and a chained equation approach method in the R MI procedure, to account for missing data. Continuous variables will be presented by mean ± standard deviation. We will use the Kolmogorov–Smirnov test to see the normality and Levene test to see homogeneity of variance. Data transformations, such as logarithmic transformation or arcsine square root transformation, will be applied in cases of a non-normal distribution. Analysis of variance (ANOVA) will be used for comparing the differences between groups, and Bonferroni test will be applied if the difference is found to be significant. Paired *t*-test will be used for comparing the differences within groups. Differences between groups of the categorical data (menstrual frequency) will be compared by Kruskal–Wallis test, and Nemenyi test will be applied if the difference is found to be significant. A two-sided *P* value less than 0.05 will be considered significant.

## Discussion

Existing pharmaceutical agents such as OCPs have been associated with side effects, and OCPs may potentially have an adverse cardiovascular risk, elevated inflammatory markers, and decreased insulin sensitivity [48]. Non-pharmacological interventions, such as aerobic exercise and *Yi Jin Jing*, have limited side effects and are widely applicable. *Yi Jin Jing* is a pleasant mind–body exercise, which is similar to other TCEs, such as Tai Chi, which has mild intensity [49]. We hypothesize that *Yi Jin Jing* and aerobic exercise could decrease AMH level and improve menstrual pattern as well as other secondary outcomes compared to control group, thus improving ovarian function of young overweight/obese women with PCOS. Obesity, insulin resistance, and metabolic disturbances are predominant in older women with PCOS; the early management of younger PCOS women might reduce their risk of insulin resistance and metabolic disturbances later in life [7]. As a result, *Yi Jin Jing* may offer an easy and inexpensive alternative management for younger women with PCOS, to further avoid long-term complications.

### Trial status

The recruitment phase has begun in July 2019 and is expected to be finished in October 2021.

## Supplementary Information

**Additional file 1:** 

## Data Availability

The authors will have access to the datasets; the datasets are not publicly available. Data will be available from the authors on reasonable request.

## Abbreviations

PCOS: Polycystic ovary syndrome
BMI: Body mass index
AMH: Anti-Mullerian hormone
SPIRIT: Standard Protocol Items: Recommendations for Interventional Trials
FSH: Follicle-stimulating hormone
E2: Estrogen
TCE: Traditional Chinese exercise
TCM: Traditional Chinese medicine
FINS: Fasting insulin level
FBG: Fasting blood glucose
FAI: Free androgen index
SHBG: Sex hormone binding globulin
T: Testosterone
DHEA-S: Dehydroepiandrosterone sulfate
ANOVA: Analysis of variance
