# Correction: Comparative effectiveness of aerobic exercise versus *Yi Jin Jing* on ovarian function in young overweight/obese women with polycystic ovary syndrome: study protocol for a randomized controlled trial

**DOI:** 10.1186/s13063-022-06547-8

**Published:** 2022-07-15

**Authors:** Jing Zhao, Antonnette Ketlhoafetse, Xiangyun Liu, Yang Cao

**Affiliations:** 1 Yueyang Hospital of Integrated Traditional Chinese and Western Medicine, Shanghai University of Traditional Chinese Medicine, 110 Ganhe Road, Shanghai, 200437 China; 2 Key Laboratory of Exercise and Health Sciences of Ministry of Education, Shanghai University of Sport, 188 Hengren Road, Shanghai, 200438 China

**Correction: Trials 23, 459 (2022)**

**https://doi.org/10.1186/s13063-022-06377-8**

Following publication of the original article [1], the authors reported an error in affiliation 1. The correct affiliation 1 has been published in this correction and given below.

1 Yueyang Hospital of Integrated Traditional Chinese and Western Medicine, Shanghai University of Traditional Chinese Medicine, 110 Ganhe Road, Shanghai 200437, China

The affiliation of Jing Zhao was incorrect upon publication. Jing Zhao is affiliated to Yueyang Hospital of Integrated Traditional Chinese and Western Medicine, Shanghai University of Traditional Chinese Medicine, 110 Ganhe Road, Shanghai 200437, China.

Further to this, the funding section has been updated as follows:

This project is funded by Shanghai '13th five-year plan' key clinical specialty (TCM gynecology department) (shslczdzk04501) and General program of clinical research of Shanghai Municipal Health Commission (202140173).

The original article [1] has been corrected.
